# Characterization of the Role of the Neoxanthin Synthase Gene *BoaNXS* in Carotenoid Biosynthesis in Chinese Kale

**DOI:** 10.3390/genes12081122

**Published:** 2021-07-24

**Authors:** Yue Jian, Chenlu Zhang, Yating Wang, Zhiqing Li, Jing Chen, Wenting Zhou, Wenli Huang, Min Jiang, Hao Zheng, Mengyao Li, Huiying Miao, Fen Zhang, Huanxiu Li, Qiaomei Wang, Bo Sun

**Affiliations:** 1 College of Horticulture, Sichuan Agricultural University, Chengdu 611130, China; 201704922@stu.sicau.edu.cn (Y.J.); 201807620@stu.sicau.edu.cn (C.Z.); 201906234@stu.sicau.edu.cn (Y.W.); 201906196@stu.sicau.edu.cn (Z.L.); 201906189@stu.sicau.edu.cn (J.C.); 201906208@stu.sicau.edu.cn (W.Z.); 201707894@stu.sicau.edu.cn (W.H.); 2018205026@stu.sicau.edu.cn (M.J.); 2020205028@stu.sicau.edu.cn (H.Z.); limy@sicau.edu.cn (M.L.); zhangf@sicau.edu.cn (F.Z.); 10650@sicau.edu.cn (H.L.); 2 Department of Horticulture, Zhejiang University, Hangzhou 310058, China; miaoamiao@zju.edu.cn

**Keywords:** Chinese kale, *BoaNXS*, carotenoid biosynthesis, subcellular localization, gene transient overexpression

## Abstract

Chinese kale (*Brassica oleracea* var. *alboglabra*) is rich in carotenoids, and neoxanthin is one of the most important carotenoids in Chinese kale. In this study, the function of the neoxanthin synthase gene (*BoaNXS*) in Chinese kale was investigated. *BoaNXS*, which had a 699-bp coding sequence, was cloned from the white flower cultivar of Chinese kale and was expressed in all developmental stages and organs of Chinese kale; its expression was highest in young seeds. The subcellular localization indicated that BoaNXS was localized in the chloroplast. *BoaNXS*-overexpressed plants were obtained via *Agrobacterium*-mediated transient overexpression methodology, and the gene overexpression efficiencies ranged from 2.10- to 4.24-fold. The color in the leaves of *BoaNXS*-overexpressed plants changed from green to yellow-green; the content of total and individual carotenoids, such as neoxanthin, violaxanthin, and lutein, was significantly increased, and the expression levels of most carotenoid biosynthetic genes were notably increased. These findings indicated that *BoaNXS* is of vital importance in carotenoid biosynthesis in Chinese kale and could be used as a candidate gene for enriching the carotenoid accumulation and color of Chinese kale and other *Brassica* vegetables.

## 1. Introduction

Carotenoids are a major group of pigments that are distributed abundantly in a variety of plants [1]. They typically contain 40 carbons in their polyene backbones with conjugated double bonds and rings at the ends [2]. They play significant roles in plant development, including light harvesting, photoprotection against excess light, and the synthesis of plant hormones [3]. Most animals obtain necessary carotenoids through their diet [4]. The accumulation of carotenoids enhances both the sensory and nutritional quality of fruits, flowers, and vegetables; carotenoids are also beneficial to human health [5]. Furthermore, they are essential precursors to several phytohormones, such as abscisic acid and strigolactones [6]. In humans, carotenoids can help prevent several major diseases, including certain cancers and eye diseases [7]. The carotenoid biosynthesis pathway in higher plants consists of the condensation of small molecular substances into C40 compounds and a series of reactions, including dehydrogenation, isomerization, cyclization, hydroxylation, and epoxidation, to produce different carotenoids [8].

Neoxanthin, which is present in light-harvesting complexes, is a precursor of abscisic acid and a xanthophyll that is the last product of carotenoid biosynthesis in green plants [9,10]. The neoxanthin synthase gene (*NXS*) encodes a 56-kDa plastid-targeted protein that, when expressed in *Escherichia coli*, catalyzes the conversion of violaxanthin to neoxanthin [9]. In plants, one of the possible pathways that is thought to underlie this conversion is as follows: violaxanthin → neoxanthin → abscisic acid [11]. Previous studies have identified *NXS* from *Arabidopsis* *thaliana* [12], tomato (*Solanum lycopersicum*) [9], and potato (*Solanum tuberosum*) [13]. However, little research has been conducted on *NXS* compared with other carotenoid biosynthetic genes.

Chinese kale (*Brassica oleracea* var. *alboglabra*) is a vegetable within the Cruciferae family native to South China, where it is distributed widely [14]. The tender leaves and bolting stems are the most commonly used edible parts because of their high content of nutrients, such as glucosinolates, vitamin C, and carotenoids [15,16]. In our previous study, four carotenoids (lutein, neoxanthin, violaxanthin, and β-carotene) were observed in Chinese kale, among which the content of neoxanthin is second only to lutein [16]. However, the synthesis of the xanthophyll neoxanthin in Chinese kale remains unclear. In this study, the full-length coding sequence (CDS) of *BoaNXS* was cloned. Sequence analysis and subcellular location were performed. The role of *BoaNXS* in carotenoid biosynthesis was characterized by phenotypic analysis, gene expression, pigment composition, and content analysis in *BoaNXS* overexpression plants.

## 2. Materials and Methods

### 2.1. Plant Materials

The cultivar ‘Sijicutiao’ of white flower Chinese kale was used in this study. The plants were grown in trays containing a mixture of peat, perlite, and vermiculite (3:1:1) in an artificial climate chamber with a light intensity of 160 μmol m^−2^ s^−1^, a temperature of 25/20 °C (day/night), a 12/12 h (day/night) light cycle, and relative humidity maintained at approximately 70%. Fertilizer and water were applied as needed. Chinese kale materials were sampled at different developmental stages (germinating seeds, cotyledons, fifth to sixth true leaves, and mature leaves), various organs were sampled at the maturity stage (roots, bolting stems, leaves, petioles, inflorescences, seed pods, and young seeds), and floral organs were sampled at the flower bud stage and the opening flower stage (sepals, petals, stamens, and pistils) [17]. The samples were then quickly frozen in liquid nitrogen and were stored in a refrigerator at −80 °C for subsequent studies.

### 2.2. Molecular Cloning and Sequence Analysis

Specific primers for the *BoaNXS* gene were designed according to the sequences of *NXS* of homologous species such as cabbage and Chinese cabbage obtained from *Brassica* database (BRAD) (http://brassicadb.org accessed on 1 May 2019) (Supplementary Table S1). The primers were synthesized by Sangon Biotech Co. Ltd. (Sangon, Shanghai, China). The cDNA of fifth to sixth true leaves was used as the template, and PCR amplification was performed using *TransStart FastPfu* fly DNA polymerase *Taq* (TransGene, Beijing, China). The method of gene cloning refers to Sun et al. [18]. The NXS amino acid sequences of other species were downloaded from NCBI (https://www.ncbi.nlm.nih.gov/ accessed on 31 July 2019) and *Brassica* database (BRAD) (http://brassicadb.org accessed on 31 July 2019) and then subjected to multiple sequence alignment using DNAMAN software (Lynnon Biosoft, Foster City, CA, USA). Subcellular localization was predicted by WoLF PSORT (http://www.genscript.com/wolf-psort.html accessed on 1 June 2019).

### 2.3. Subcellular Localization

The complete CDS of *BoaNXS* was amplified by primers *NXS*-GFP-F and *NXS*-GFP-R (Table S1) containing *BamH* I and *Sal* I restriction sites. Then, BoaNXS and pC2300-35S-eGFP plasmid digested with *BamH* I and *Sal* I were mixed to construct the pC2300-35S-NXS-eGFP plasmid. The recombinant plasmid pC2300-35S-NXS-eGFP and empty vector pC2300-35S-eGFP were transformed into Chinese kale mesophyll protoplasts, respectively. After being cultured in the dark at 23 °C for 24 h, the protoplasts were observed using a BX51 fluorescence microscope equipped with a DP70 camera (Olympus, Tokyo, Japan) [19].

### 2.4. Transient Overexpression of BoaNXS

Transient overexpression assay was conducted using the methods of a previous study [20]. The complete CDS of *BoaNXS* was amplified by primers *NXS*-pCAM-F and *NXS*-pCAM-R (Supplementary Table S1) containing *BamH* I and *Sal* I restriction sites. Then, BoaNXS and pCAMBIA1301-35S-Nos overexpression vector (kindly supplied by Associate Professor Chen Qing, Sichuan Agricultural University, China) digested with *BamH* I and *Sal* I were mixed to construct the pCAMBIA1301-BoaNXS plasmid. The recombinant plasmid pCAMBIA1301-BoaNXS and empty vector pCAMBIA3101 were transferred into *Agrobacterium* GV3101 by freeze-thaw method, and positive clones were screened under kanamycin, gentamicin, and rifampicin resistance conditions.

The *Agrobacterium* strains were washed with the infiltration buffer (10 mmol L^−1^ MES, 0.2 mmol L^−1^ acetosyringone, 10 mmol L^−1^ MgCl_2_) and were then cultured to OD600 = 0.6~0.8. The 4-week-old plants of Chinese kale cultivar ‘Sijicutiao’ were treated by using the infiltration buffer containing pCAMBIA1301-BoaNXS construct or pCAMBIA1301 empty vector, and ddH_2_O was used as the control. The second and third true leaves were injected until the liquid could fully penetrate the leaf back, and were then injected by using a needleless syringe to infiltrate the *Agrobacterium* every week, four times in total. The second day after the last injection, the leaves were sampled to detect the gene expression and carotenoid contents.

### 2.5. Color Analysis

Color analysis was performed on the leaves of overexpressed and control plants using an NR110 chromameter (3nh, Shenzhen, China). Three positions were randomly selected on the sampled leaves of each plant, and the values of L*, a*, and b* were statistically analyzed. L* values represented the lightness of the color, ranging between black (L* = 0) and white (L* = 100), a* values represented the color’s position between green (negative a*) and red (positive a*), and b* values represented the color’s position between blue (negative b*) and yellow (positive b*).

### 2.6. Determination of Carotenoid and Chlorophyll Composition and Contents

Carotenoid and chlorophyll concentrations were determined using the methods of a previous study [16]. Two hundred milligrams of leaves were ground and extracted with 25 mL of acetone, and were sonicated for 20 min and then centrifuged at 4000× *g* at room temperature for 5 min. The supernatant was filtered through 0.22 μm cellulose acetate filters and then analyzed by high-performance liquid chromatography (HPLC). HPLC analysis of carotenoids was carried out using an Agilent 1260 instrument equipped with a variable wavelength detector (VWD). Samples (10 μL) were separated at 30 °C on a Waters Nova-Pak C18 column (150 × 3.9 mm id; 3 μm particle size) using isopropanol and 80% acetonitrile-water at a flow rate of 0.5 mL min^−1^; the absorbance was measured at 448 and 428 nm (Supplementary Figure S1). Carotenoids (neoxanthin, violaxanthin, lutein, and β-carotene) and chlorophyll (chlorophyll a and chlorophyll b) were quantified according to the respective standard calibration curves, and their standards were obtained from Solarbio Science & Technology Co., Ltd. (Beijing, China).

### 2.7. RNA Extraction and qPCR Analysis

Total RNA was extracted using an alternative CTAB method. RNA concentration and quality were determined by photometric measurements (General Electric Company, Schenectady, NJ, USA) and gel electrophoresis. Intact total RNA was used for cDNA synthesis using the PrimeScript™ 1st Strand cDNA Synthesis Kit (TaKaRa, Dalian, China).

The quantitative real-time PCR (qPCR) primers for carotenoid biosynthetic genes in Chinese kale were synthesized according to a previous study [16], and *β-actin* was used as the reference gene (Table S1). The expression of *BoaNXS* and other carotenoid biosynthetic genes (*PSYs*, *PDSs*, *ZISO*, *ZDS*, *CRTISO*, *LCYb*, *LCYes*, *β-OHase*, *ε-OHase*, *VDE*, *ZEPs*) [21] of overexpressed plants and control plants were performed by using the Bio-Rad iCycler thermocycler (Bio-Rad, Hercules, CA, USA), and the 2^−ΔΔCT^ method was used to calculate the gene expression levels [22].

### 2.8. Data Analysis

The results were shown as the mean ± standard deviation (SD) of the three technical replicates. Statistical analysis was performed with SPSS software package 18 (SPSS Inc., Chicago, IL, USA). One way analysis of variance (ANOVA) was used to analyze the data. The least significant difference (LSD) test was used to compare the differences at the significance level of 0.05.

## 3. Results

### 3.1. Isolation and Characterization of BoaNXS

**Figure 1:**
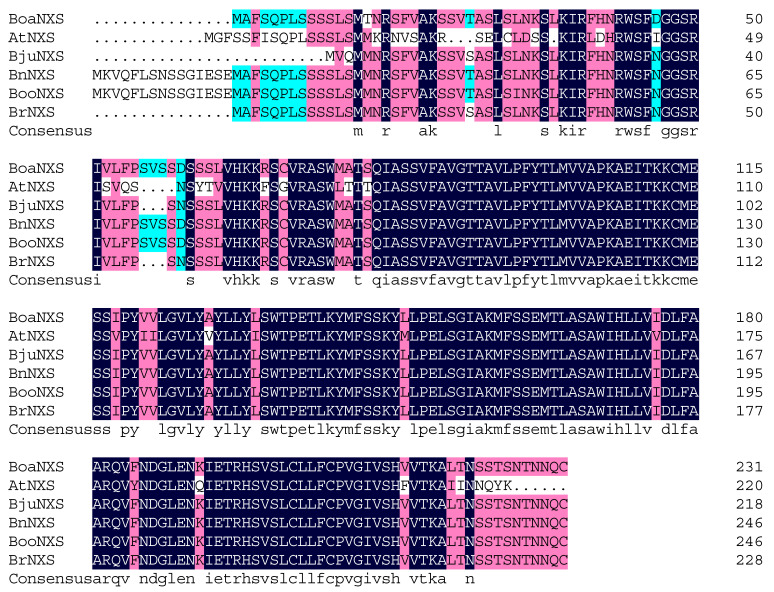
Multiple sequence alignment of NXS from different plants. Alignment of the protein sequence of BoaNXS with selected homologs. The alignment was performed using DNAMAN software. The amino acids with 100% identity are shown with a black background, those with ≥75% identity are shown in red, and those with ≥50% identity are shown in blue. The species and their accession numbers in GenBank (*Brassica oleracea* var. *alboglabra* (Boa): BoaNXS (MW284390), *Arabidopsis thaliana* (At): AtNXS (VYS50248.1), *Brassica juncea* (Bju): BjuNXS (BjuA007212), *Brassica napus* (Bn): BnNXS (XP_013676374.1), *Brassica oleracea* var. *oleracea* (Boo): BooNXS (XP_013618475.1), *Brassica rapa* (Br): BrNXS (XP_009127521.1) are listed here.

The CDS of *BoaNXS* was cloned from Chinese kale leaves and had a sequence length of 699 bp (GenBank accession MW284390), which encoded a 232-amino acid protein and belonged to the DUF4281 superfamily. The multiple sequence alignment results showed that the changes in the amino acid sequences of NXS among different species were mainly located at the N-terminus (Figure 1).

### 3.2. Temporal and Spatial Expression of BoaNXS

**Figure 2:**
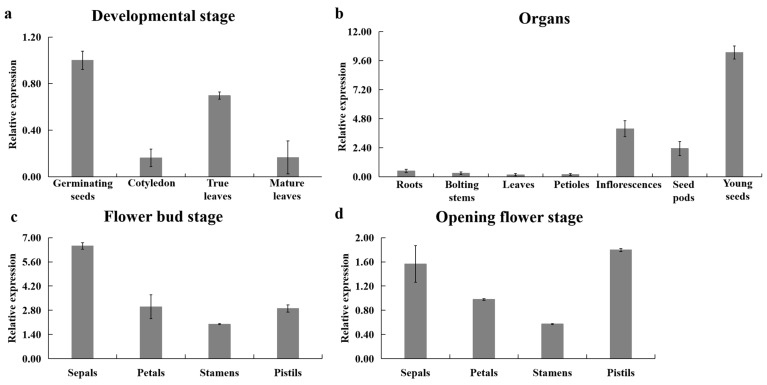
Relative expression levels of *BoaNXS* in different developmental stages (**a**), organs (**b**), and flower tissues in flower bud stage (**c**) and opening flower stage (**d**) of Chinese kale. The expression level of *BoaNXS* in germinating seeds was set as 1.

The temporal and spatial expression results showed that *BoaNXS* was detected at all developmental stages and organs in Chinese kale (Figure 2). During the development of Chinese kale, the highest level of *BoaNXS* expression was in the germination stage, followed by the true leaf stage; *BoaNXS* expression was lowest in the cotyledon and maturity stages (Figure 2a). Among different organs at the maturity stage, the highest expression level of *BoaNXS* was observed in young seeds, where *BoaNXS* expression was twice that observed in other organs, followed by inflorescences and seed pods; relatively low expression was observed in the roots, bolting stems, petioles, and leaves (Figure 2b). Among different flower tissues, the lowest expression level of *BoaNXS* was observed in stamens. Furthermore, the expression levels of *BoaNXS* in all flower tissues decreased from the flower bud stage to the opening flower stage (Figure 2c,d).

### 3.3. BoaNXS Was Localized in the Chloroplast

**Figure 3:**
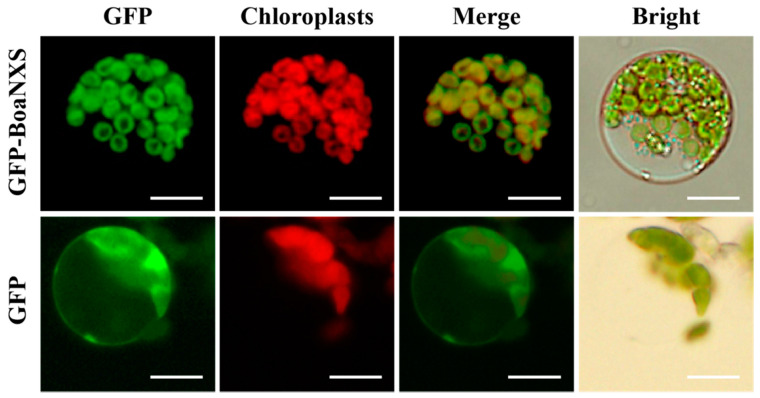
Subcellular localization of BoaNXS in Chinese kale. Free green fluorescing protein served as a control. The upper column represents transient expression of GFP-BoaNXS fusion protein in Chinese kale protoplasts, and the lower column represents transient expression of GFP protein in Chinese kale protoplasts. Merge represents the merged images of GFP (green) and chloroplast autofluorescence (red). Bars = 30 μm.

WoLF PSORT software predicted that BoaNXS was most likely to be located in the chloroplast. A clear GFP fluorescence signal of BoaNXS was only detected in the chloroplast (Figure 3), and the expression of the GFP protein was detected in the entire protoplast with green fluorescence signal in the control. These results indicated that BoaNXS was specifically localized in the chloroplast, which was consistent with the software prediction.

### 3.4. BoaNXS Overexpression Affected the Color of Chinese Kale

**Figure 4:**
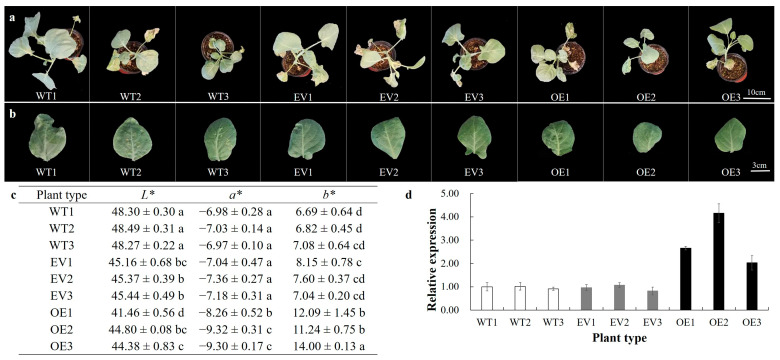
The phenotypes and expression of *BoaNXS* in wild-type (WT), the agroinfiltrated plants with empty vector (EV), and the overexpressed plants (OE) of Chinese kale: (**a**) Top view of the control (WT), EV, and OE plants. Bar = 10 cm; (**b**) The leaf front of sampling leaves. Bar = 3 cm; (**c**) The color parameters of the control (WT), EV, and OE plants at the second day after the last injection. L* values represent the lightness of the color, a* values represent the color’s position between green and red, and b* values represent the color’s position between blue and yellow. Data are expressed as a mean ± standard deviation (SD) of three replicates. Different lowercase letters in the same column indicate significant differences among values (*p* < 0.05) according to a Least Significant Difference (LSD) test; (**d**) The relative expression levels of *BoaNXS* in the control (WT), EV, and OE plants at the second day after the last injection.

*BoaNXS*-overexpressed plants were yellow-green compared to the dark-green of the control (the wild-type (WT) plants) and agroinfiltrated plants with empty vector (EV) (Figure 4a,b). The red-green value a* was significantly lower for *BoaNXS*-overexpressed plants than for control and EV plants (Figure 4c), indicating that the leaves of the overexpressed plants were greener. Moreover, the yellow-blue value b* was significantly lower for *BoaNXS*-overexpressed plants than for control and EV plants (Figure 4c), indicating that the leaves of the overexpressed plants were yellowing. The *BoaNXS* gene expression levels of OE plants were 2.10- to 4.24-fold higher than those in WT and EV plants, especially in OE2 (Figure 4d).

### 3.5. Overexpression of BoaNXS Increased Carotenoid Accumulation in Chinese Kale

**Figure 5:**
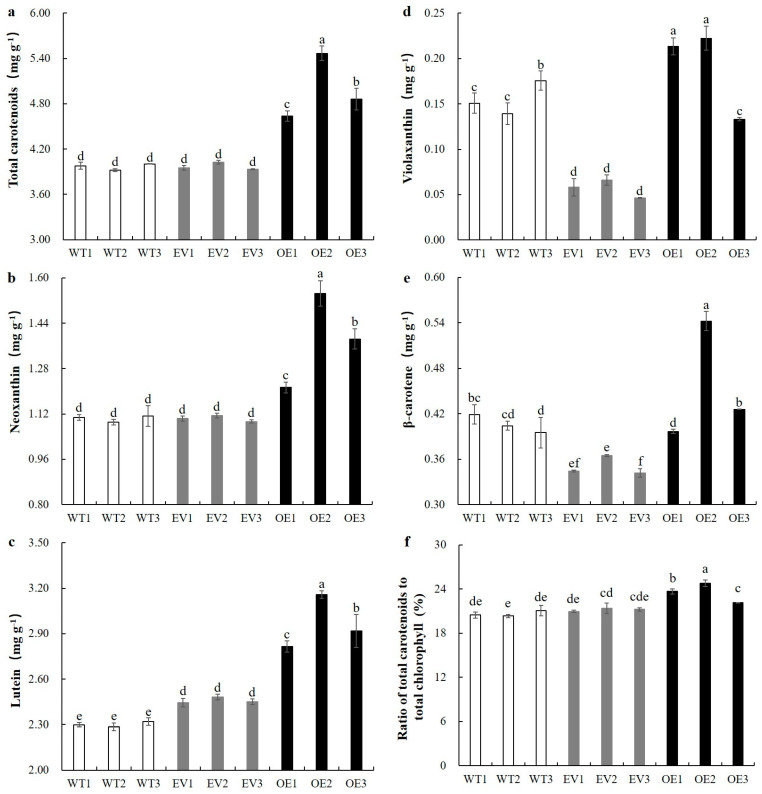
Individual and total carotenoid content and ratio of total carotenoids to total chlorophyll of wild-type (WT), the agroinfiltrated plants with empty vector (EV), and the *BoaNXS*-overexpressed plants (OE) in Chinese kale. (**a**) content of total carotenoids; (**b**) neoxanthin content; (**c**) lutein content; (**d**) violaxanthin content; (**e**) β-carotene content; (**f**) ratio of total carotenoids to total chlorophyll. Samples of leaves were taken from the control (WT), EV, and OE plants at the second day after the last injection. Data are expressed as mean ± standard deviation. Different letters above the bars indicate significantly different values (*p* < 0.05) according to a Least Significant Difference (LSD) test.

The content of total carotenoids was significantly increased in all three overexpressed plants (OE1, OE2, and OE3) (Figure 5a). The average content of total carotenoids in overexpressed plants was 4.99 mg g^−1^ DW, which was 1.26-fold higher compared with the average content of the control plants. Four carotenoids were detected in Chinese kale leaves: neoxanthin, violaxanthin, lutein, and β-carotene. The content of neoxanthin and lutein was significantly higher in all three overexpressed plants than in both WT and EV plants (Figure 5b,c), whereas violaxanthin content in OE1 and OE2 leaf tissues (Figure 5d), as well as β-carotene content in OE2 leaf tissue (Figure 5e), was significantly higher compared with that in WT plants. Although there were significant differences in the content of several individual carotenoids (violaxanthin, lutein, and β-carotene) between WT and EV plants, the content of total carotenoids did not significantly differ between WT and EV plants. Among the overexpressed plants, OE2 leaf tissue had the highest total and individual carotenoids (neoxanthin, violaxanthin, lutein, and β-carotene), which were up to 1.38, 1.40, 1.38, 1.37, and 1.32-fold higher compared with the average level of WT plants, respectively. The ratio of total carotenoids to total chlorophyll is also shown in Figure 5f, and the results are basically consistent with the color analysis results (Figure 4).

### 3.6. Overexpression of BoaNXS Induced the Expression of Carotenoid Biosynthetic Genes

**Figure 6:**
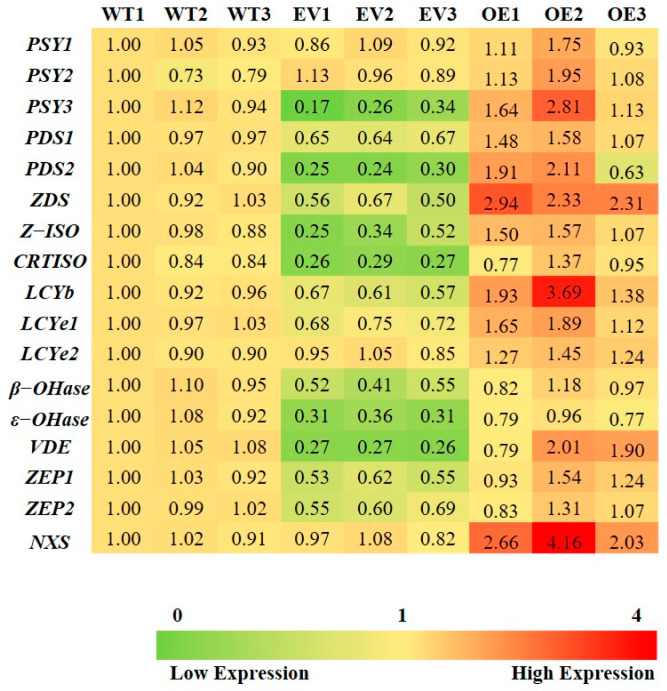
Heat map of carotenoid biosynthetic gene expression in wild-type (WT), the agroinfiltrated plants with empty vector (EV), and the *BoaNXS*-overexpressed plants (OE) in Chinese kale. Samples of leaves were taken from the control (WT), EV, and OE plants at the second day after the last injection. Abbreviations: GGPP, geranylgeranyl diphosphate; *PSY*, phytoene synthase; *PDS*, phytoene desaturase; *ZDS*, ζ-carotene desaturase; *Z-ISO*, ζ-carotene isomerase; *CRTISO*, carotenoid isomerase; *LCYe*, lycopene ε-cyclase; *LCYb*, lycopene β-cyclase; *ε-OHase*, ε-carotene hydroxylase; *β-OHase*, β-carotene hydroxylase; *VDE*, violaxanthin de-epoxidase; *ZEP*, zeaxanthin epoxidase; *NXS*, neoxanthin synthase.

To determine whether *BoaNXS* affects carotenoid accumulation by regulating the transcription of other carotenoid biosynthetic genes, its expression in WT, EV, and OE leaf tissues were analyzed using qPCR (Figure 6). The average expression levels of all carotenoid biosynthetic genes in OE leaf tissues were notably increased, except for *β-OHase* and *ε-OHase*, which showed a slight decline. With the exception of *BoaNXS*, the average expression level of *ZDS* in OE leaf tissues was the highest among carotenoid biosynthetic genes in Chinese kale, which was 2.57-fold higher compared with WT plants. In addition, the average expression levels of most carotenoid biosynthetic genes in EV plants were lower compared with WT plants. These findings suggest that the up-regulation of *BoaNXS* expression promotes the entire carotenoid biosynthetic pathway in Chinese kale.

## 4. Discussion

By bringing *Agrobacterium* into contact with the recipient material through vacuum infiltration, floral dip, or clinical syringe injection methods, *Agrobacterium*-mediated transient expression is an effective tool for exploring the function of candidate genes in plants [23]. Transient expression technology has several advantages over stable transformation, such as being rapid and simple to perform [24]. In this study, we successfully overexpressed *BoaNXS* in Chinese kale via transient expression technology, which has been rarely used in research on *Brassica* vegetables. We found that the expression levels of *BoaNXS* in overexpressed plants were higher than those in WT plants and agroinfiltrated plants with empty vector. Thus, the *Agrobacterium*-mediated transient overexpression technology in this study could facilitate the study of gene function in Chinese kale and other *Brassica* vegetables. In previous studies, transient overexpression efficiencies of rice (*Oryza sativa* L. subspecies *indica*), banana (*Musa* sp.), and *Salvia miltiorrhiza* were 4.1~7.8 [25], 2.12~4.93 [26], and 3.77~8.39-fold [27], respectively. The gene overexpression efficiencies ranged from 2.10- to 4.24-fold in our study, which is lower compared with some of the above values. In future experiments, we plan to improve the efficiency of transient overexpression by increasing the number of injections, adjusting the sampling time, and changing the strain of *Agrobacterium*. Simultaneously, stable transformation could be used to verify the results.

The leaves of Chinese kale turned yellow, and the b* value increased in overexpressed plants. This result was similar to previous results in other carotenoid synthetic genes. For example, potato tubers overexpressing *StLCYb* appeared yellow; in contrast, the control plant was light-white [28]. Canola seeds (*Brassica napus*) overexpressing *crtB* (a bacterial phytoene synthase gene) were visibly orange [29]. In addition to leaves, tubers, and seeds, color changes have also been observed in floral organs. For example, *Iris germanica* L. ‘Fire Bride’ ectopically expressing *crtB* exhibited pronounced color changes in the ovaries (green to orange), flower stalk (green to orange), and anthers (white to pink) [30]. In our study, the expression of *BoaNXS* was clearly higher in the flower bud stage of Chinese kale than in the opening flower stage, and this finding was similar to the result of a previous study of Tiger Lily, in which the transcript levels of *LllcyB* decreased as flower buds developed [31].

NXS catalyzes the formation of neoxanthin from violaxanthin. When *T.NXS* was transiently overexpressed in tobacco leaves, the neoxanthin content increased as the content of violaxanthin decreased, and there was no significant change in the total pigment content [9]. Our result was inconsistent with this previous study but consistent with a previous study of *Arabidopsis* [12], in which the overexpression of *BoaNXS* resulted in consistent increases in both the neoxanthin and violaxanthin content compared with the control. This increase might stem from feedback control on an isomerase, which acts on both *trans*-neoxanthin and *trans*-violaxanthin [12], based on the increased levels of *trans*-neoxanthin generated from higher BoaNXS activity. This self-perpetuating state can promote increased carotenoid synthesis, so we speculate it could be regulated by a positive feedback mechanism [32]. Similarly, overexpression of *Dclcyb1* in tobacco increased β-carotene, lutein, and total carotenoids [33]; overexpression of the *IbZDS* gene in agroinfiltrated sweet potato increased β-carotene and lutein content [34]; and up-regulation of an endogenous *PSY* gene in *Arabidopsis* increased β-carotene, lutein, violaxanthin, and the total carotenoid levels [35]. In our study, overexpression of *BoaNXS* increased the content of individual and total carotenoids, which indicated that this is an effective method for improving the variety of Chinese kale. This further indicates that *BoaNXS* plays an important role in the regulation of carotenoid content in Chinese kale. Furthermore, the expression of *BoaNXS* greatly increased the accumulation of transcripts of most carotenoid biosynthetic genes in OE leaf tissues (Figure 6). The same effect was observed in carrot where transcripts of endogenous *Dcpsy1*, *Dcpsy2*, and *Dclcyb2* genes were increased when *Dclcyb1* was overexpressed [36]. These results indicated that overexpression of *BoaNXS* induced a positive feedback loop affecting the expression of genes involved in carotenoid biosynthesis. Some carotenoids contents and pathway genes were downregulated in EV plants compared with the control in our results, which means the infection of *Agrobacterium* might affects the carotenoid biosynthesis. Nevertheless, its specific mechanism is still unclear, and the cause of this phenomenon needs further study.

Carotenoid enzymes are found in plastids (especially in chloroplasts and chromoplasts) of plant cells [37], where they exert their functions. *BoaNXS* translation forms neoxanthin synthase. Neoxanthin synthase is a class of important carotenoid enzymes that participate in carotenoid biosynthesis, ABA synthesis, and photosynthesis [38,39]. Abscisic acid (ABA) is derived from the cleavage of 9-cis isomers of violaxanthin and neoxanthin [38] and functions in embryogenesis and seed germination [40]. This could explain why the expression levels of *BoaNXS* were the highest in young seeds compared with other organs in our study and indicates that *BoaNXS* might be related to reproductive growth. In our following experiments, we will also focus on the effects of *BoaNXS* on ABA biosynthesis and measure some ABA biosynthetic genes to further explore the relationship between *BoaNXS* and ABA biosynthesis. Dall’Osto et al. found that neoxanthin played a specific role in the protection of Lhc proteins, the photosystem II (PSII) reaction center, and thylakoids from photooxidative stress and that its action was effective against the damaging effect of reactive oxygen species, particularly superoxide anion [39].

## 5. Conclusions

In summary, the neoxanthin synthase gene *BoaNXS* was cloned, and BoaNXS was located in the chloroplast. The expression of *BoaNXS* was detected in all development stages and organs of Chinese kale. *BoaNXS*-overexpressed agroinfiltrated plants were obtained through *Agrobacterium*-mediated transient expression technology. The induced expression of *BoaNXS* and most other carotenoid biosynthetic genes, as well as the increased carotenoid content, indicated that *BoaNXS* plays an important role in carotenoid biosynthesis in Chinese kale.

## Data Availability

The data presented in this study are available in the manuscript and supplementary material.

## Supplementary Materials

The following are available online at https://www.mdpi.com/article/10.3390/genes12081122/s1, Figure S1: High-performance liquid chromatography profile of carotenoids and chlorophyll in Chinese kale leaves. Table S1: Primers used in this study.
