# Supplementary material for: Characterization of the Role of the Neoxanthin Synthase Gene BoaNXS in Carotenoid Biosynthesis in Chinese Kale
Source: Genes (Basel). 2021 Jul 24;12(8):1122. doi: 10.3390/genes12081122 (PMC8393425; doi:10.3390/genes12081122)
Supplement: Supplementary file 1 [file genes-12-01122-s001.zip › genes-1271083-SI.pdf]

Table S1 Primers used in this study

Primer name	Primer sequence (5'-3')	Aims
NXS-F	ATGGCTTTTTCTCAGCCTTTGTCTTC	Molecular cloning of *NXS* CDS
NXS-R	TTACTTGCACTGGTTGTTGGTGTTG	
NXS-GFP-F	CGGGATCCATGGCTTTTTCTCAGCCTTTGTCTTC	Subcellular localization
NXS-GFP-R	GCGTCGACCTTGCACTGGTTGTTGGTGTTG	
NXS-pCAM-F	CGGGATCCATGGCTTTTTCTCAGCCTTTGTCTTC	Primers for overexpression sites
NXS-pCAM-R	GCGTCGACCTTGCACTGGTTGTTGGTGTTG	
NXS-qPCR-F	CAGTTGACATGCCAGCAAGTCC	Detection of gene expression
NXS-qPCR-R	CGAATCGGATGATACACTGGGGA	
β-actin-qPCR-F	CCAGAGGTCTTGTTCCAGCCATC	
β-actin-qPCR-R	GTTCCACCACTGAGCACAATGTTAC	
PSY1-qPCR-F	AAGGGCTGTAGAGTCTTCTAGA	
PSY1-qPCR-R	CGTTTTTGTTGTTTGCTTCCTC	
PSY2-qPCR-F	GGGACCTTGATGATCTGAAGAA	
PSY2-qPCR-R	TTCACCCAACAAACTCAAACTC	
PSY3-qPCR-F	TTCATGAGACTGCAGCTTAAGA	
PSY3-qPCR-R	CCCAACATAAGCTCTCTTGGTA	
PDS1-qPCR-F	GATCTCTTCACAAGCGCTTAAG	
PDS1-qPCR-R	GCTTCCAAGAAATTGACAGTGT	
PDS2-qPCR-F	TCATCTGGAGGTTGTGATTTGA	
PDS2-qPCR-R	ATATCCACACAAACTACCTGCA	
ZISO-qPCR-F	CGTTGTTTGGATCGATAACTCC	
ZISO-qPCR-R	GCTAATCCACTATGCACAGTTG	
ZDS-qPCR-F	CGTTCCCTTGTCCCGAGCAA	
ZDS-qPCR-R	CCTCGGAGGTTTCATGTTAGGTCTTC	
CRTISO-qPCR-F	AGATTGGGAGGGACTCACTCCA	
CRTISO-qPCR-R	CGTTCCCTTGTCCCGAGCAA	
LCYb-qPCR-F	GTTGTTGATCTAGCTATCGTTGGC	
LCYb-qPCR-R	GAGTTTGGGGGAAGGATCGAT	
LCYe1-qPCR-F	GGTTTGTGTAGTAGAGTCGTCA	
LCYe1-qPCR-R	TCAACGAGCTTAGACTGTTCAT	
LCYe2-qPCR-F	CAGGTTCCGGTATAGAGAGTTG	
LCYe2-qPCR-R	ACGTATAGAATCTCCGAACCAC	
β-OHase-qPCR-F	CCTAATGGAGTGAAAAGCATCG	
β-OHase-qPCR-R	TCTTTACTGTTGATGGGAAGCT	
ε-OHase-qPCR-F	TCTCCTAAACCCAGATTCGTCTCC	
ε-OHase-qPCR-R	GCGAGTGAGTGATGTGAGCCA	
VDE-qPCR-F	AAGGCATTTCCTCACATCCTTA	

VDE-qPCR-R	ACCAAGAAAGTGCCTTTGATTC
ZEP1-qPCR-F	AGAAGTCCTAGTTTCACTTGGG
ZEP1-qPCR-R	TCTGTTTCCATGCTTGTTCAAG
ZEP2-qPCR-F	AGAACTCCTAGTTTCACTTGGG
ZEP2-qPCR-R	TATCCTCAATGGCCATACATCC

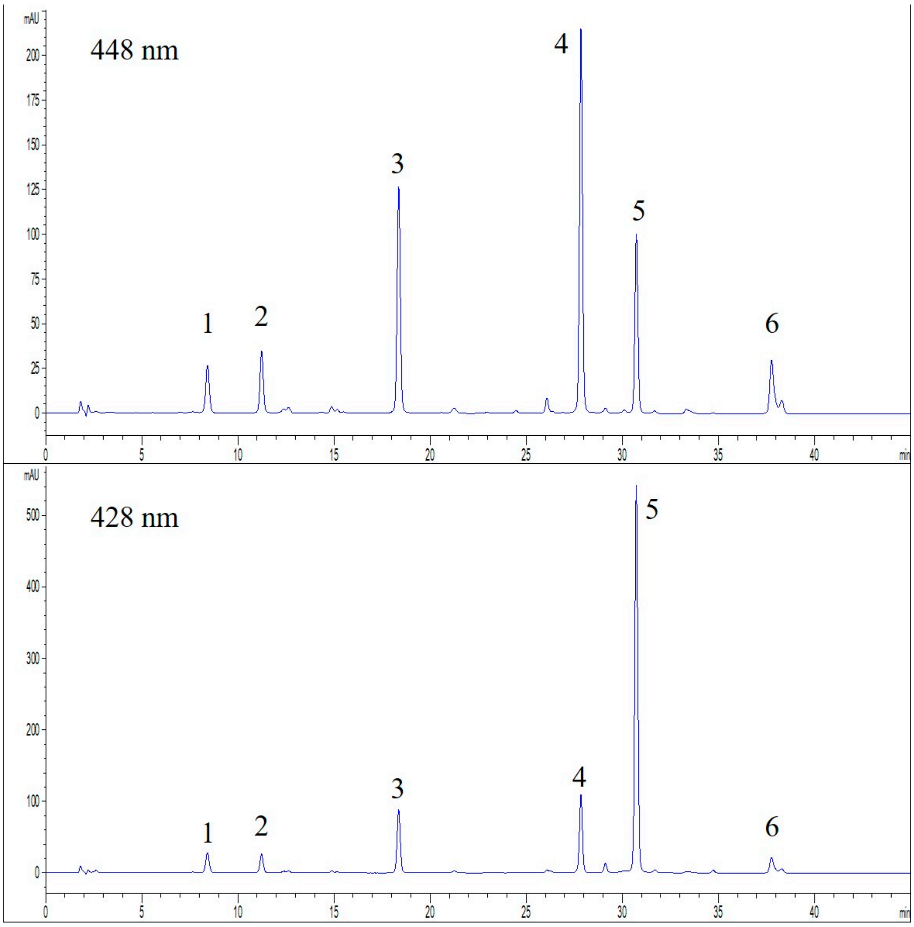

Figure S1. High-performance liquid chromatography profile of carotenoids and chlorophyll in Chinese kale leaves. 1, neoxanthin, 2, violaxanthin, 3, lutein, 4, Chlorophyll b, 5, chlorophyll a, and 6, β-carotene. Chlorophyll a is detected at 428 nm, and other pigments are detected at 448 nm.
